# Clinical characteristics and outcomes of referrals for speech and language delay to the Speech Therapy and Audiology department at a district hospital in Gauteng, South Africa

**DOI:** 10.4102/sajcd.v73i1.1153

**Published:** 2026-08-22

**Authors:** Lucie B.L. Graca, Renate Strehlau, Jacqueline K. Bezuidenhout

**Affiliations:** 1 Department of Paediatrics and Child Health, Faculty of Health Sciences, University of the Witwatersrand, Johannesburg, South Africa

**Keywords:** speech delay, language delay, hearing loss, autism spectrum disorder, speech therapists and audiologists, early referrals and interventions, district hospital, hearing impairment

## Abstract

**Background:**

Speech and language skills are essential for effective communication. Speech and language delays may have far-reaching consequences on a child’s development; their early identification and intervention are paramount to a child reaching their full potential.

**Objectives:**

To describe the referral process, clinical characteristics, risk factors and outcomes of paediatric referrals to a Speech Therapy and Audiology (STA) department at a district-level hospital in Gauteng, South Africa.

**Method:**

A retrospective record review was conducted on paediatric outpatient referrals to the STA department between the 01 January 2021 and 30 June 2022. Descriptive statistical analysis was performed on the categorical data. Associations between receptive and expressive language delays and the presence of maternal comorbidities or a bilingual household were analysed using the Chi-squared test and Fisher’s exact test.

**Results:**

A total of 150 referrals were reviewed, with speech delay being the most common reason for referral (38%). Children under 2 years old accounted for 42% of the referrals. Of the children seen by the speech therapists and audiologists, 58 (49.2%) defaulted on follow-up appointments, and three (2.5%) were identified as having hearing loss. Twenty-eight children (23.7%) were referred for neurodevelopmental assessments, of whom 52% received a diagnosis of autism spectrum disorder (ASD). The mean age of ASD diagnosis was 49.54 months (standard deviation 11.47).

**Conclusion:**

Understanding the clinical profile and outcomes of children referred to a STA department at a district-level hospital provides valuable insight into the vital role of the speech therapists and audiologists in early identification and intervention of children with speech and language delay.

**Contribution:**

The STA departments at South African district hospitals are well-positioned for early identification and intervention, particularly for neurodevelopmental conditions, including ASD.

## Introduction

Age-appropriate speech and language development is an essential skill for children to master, ensuring effective communication, participation in daily activities and the development of literacy and numeracy skills.

Failure to attain speech and language competency may lead to learning difficulties and scholastic failures, as well as limited future employment opportunities, which may result in financial challenges in adulthood (Vameghi et al., 2015; Van Der Linde et al., 2016).

Speech is the oral-motor act of sound production and word construction; receptive language is the understanding of language and expressive language allows for the expression of thoughts, ideas and feelings (McLaughlin, 2011). The first 3 years of life are a critical period for speech and language development. It is imperative that children with communication difficulties be identified during this early developmental stage and referred for assessment and early interventions to avoid irreversible repercussions. A speech or language delay refers to a developmental condition where the sequence of the child’s speech and language acquisition is typical; however, the rate of acquisition is slower than expected as per the child’s developmental age (Wallace et al., 2015). The prevalence of speech and language delay varies across regions and is estimated to be just under 8% in the United States (US) (Black et al., 2015). Data from low- and middle-income countries (LMICs) report higher prevalence rates, with 13% – 29.1% of children under the age of 3 years having speech and language delays (Mondal et al., 2016; Van Der Linde et al., 2016; Zaib et al., 2022).

Postulated reasons for the higher prevalence in LMICs include exposure to multiple risk factors, such as poverty, limited healthcare services and residential density, in particular, the number of siblings in the household (Van der Linde et al., 2009, 2016). Other risk factors for speech and language delay, which have been reported in both high-income countries and LMICs, include male gender; lower maternal educational attainment; a family history of speech and language delay; and perinatal risk factors, including prematurity, low birth weight, birth complications and the need for admission to neonatal intensive care (Uzun Çiçek et al., 2020; Wallace et al., 2015).

Impairments in language development are often the primary presentation in children with neurodevelopmental conditions, including autism spectrum disorder (ASD). Autism spectrum disorder is a neurodevelopmental condition characterised by deficits in social communication, repetitive behaviours and/or restrictive interests, with a hierarchical severity level (American Psychiatric Association, 2022). The prevalence of ASD is increasing, with a recent publication reporting an estimated prevalence in the US of 32.2 per 1000 children aged 8 years of age (Shaw et al., 2025). As a result of the paucity of data from LMICs, the exact prevalence of ASD in these settings is largely unknown. However, studies from Uganda and Nigeria reported a prevalence of 0.68% and 2.3%, respectively (Kakooza-Mwesige et al., 2014; Lagunju et al., 2014). The Nigerian cohort had a mean age at diagnosis of 44.9 (standard deviation [s.d.] = 21.1) months and the presenting symptom in 52 (96.3%) of the children diagnosed with autism was delayed speech (Lagunju et al., 2014). Other developmental conditions often presenting with speech and language delays are children with attention deficit hyperactivity disorder (ADHD) and global developmental delay (GDD). The diagnosis of ADHD is made when the individual presents with features of inattention; hyperactivity-impulsivity, or a combination of both, for at least 6 months (American Psychiatric Association, 2022). Global developmental delay is described in children under the age of 5 years who present with significant delays in two or more developmental domains (American Psychiatric Association, 2013).

Hearing loss, often referred to as the ‘silent epidemic’, is another important medical cause of speech and language delay (Kanhere & Sunderajan, 2019). In 2018, the World Health Organization estimated 466 million individuals globally to be living with disabling hearing loss, with sub-Saharan Africa identified as one of the most affected areas (WHO, 2018). Furthermore, a worrisome 34 million of these individuals were children (Kuschke et al., 2020). Hearing plays a vital role in speech development and language acquisition, and the consequences of hearing loss, especially when identified late, can severely impact a child’s developmental potential (Lieu et al., 2020).

Screening programmes play a critical role in ensuring early detection and appropriate referrals for improved long-term health outcomes. In South Africa, the Road to Health Booklet (RtHB) is a readily accessible screening tool in primary healthcare (PHC) facilities, which can be used to identify hearing impairment and speech and language delay (Du Plessis et al., 2017). This subjective measure enables healthcare workers at PHC facilities to identify children with possible developmental delays and refer families for further assessments at district, regional or tertiary facilities (Du Plessis et al., 2017). The consistent use of standardised screening tools plays a vital role in conducting population-wide assessments to identify children in need of formal assessment.

The Speech Therapy and Audiology (STA) department at a district hospital in the South African government healthcare sector is ideally placed to streamline appropriate referrals from PHC clinics for further specialist assessment, while simultaneously initiating early intervention. Currently, limited data are available on STA referral outcomes at district-level facilities in South Africa. We conducted an audit of children referred to an outpatient STA department at a district-level hospital in an urban South African setting with the aim of describing the referral process, clinical characteristics, risk factors and outcomes of the referrals. The audit’s findings may assist in better understanding the health needs of the referred children and the utilisation of the STA department services by the PHC facilities.

## Research methods and design

The aim of the study was to audit referrals of children less than 6 years of age to the STA department at a district-level hospital in Gauteng, South Africa, with respect to the referral process, clinical characteristics, risk factors and outcomes. We also sought to identify any associations between language delay and relevant risk factors. The study utilised a retrospective record review to audit paediatric outpatient referrals to the STA department at South Rand Hospital (SRH) from 01 January 2021 to 30 June 2022. The audit was conducted in the STA department at SRH, a district-level state hospital in Rosettenville, south of Johannesburg. It is positioned as an intermediary between the PHC clinics and specialised secondary and tertiary hospitals. It thus receives referrals from surrounding clinics, hospitals, schools and other therapeutic services. The STA department receives both inpatient and outpatient referrals for children under 6 years of age. South Rand Hospital does not offer any specialist or subspecialist services; however, a neurodevelopmental clinic (NDC) is held monthly, led by a neurodevelopmental paediatrician. The NDC is an interdisciplinary setting where the visiting neurodevelopmental paediatrician, senior medical officer, speech therapists and occupational therapists from the facility discuss and assess the children with suspected developmental disorders, thereby ensuring a cohesive individualised care plan once the diagnosis has been made.

Data were collected over an 18-month period from 01 January 2021 to 30 June 2022. Historical referral records indicated the STA department receives 8–12 new patient referrals per month. Using the published prevalence data from Van der Linde et al. (2016) of 13%; a confidence interval of 95%; a 3% margin of error; and a known finite population, the sample size was calculated at 111 for the lower referral estimate and 149 for the higher estimate. The study timeframe coincided with the coronavirus disease 2019 (COVID-19) pandemic, during which services offered by the STA department were considered non-essential and therefore intermittently suspended. However, the target sample size was still achieved.

The data variables collected included the patient’s demographic details; date, source and reason for referral; perinatal history; family history of language delay; relevant past medical history; speech and language assessment results; audiological assessment results; subsequent referral requirements; date seen at the NDC and the final diagnosis; as well as the presence of syndromes. The referral outcomes of children post-assessment at the STA department were also documented.

Descriptive summary statistics were employed to analyse categorical and numerical variables. Categorical data were tallied into frequency counts and percentages, which were then tabulated and presented. Chi-squared and Fisher’s exact test were used to measure associations between language delay and living in a bilingual household, as well as language delay and the presence of maternal comorbidities. The results were expressed as odds ratios (OR) and *p*-values. Statistical analysis was performed in Microsoft Excel, and a *p*-value of < 0.05 was considered significant.

Data reliability was ensured by collecting pre-set, clearly defined variables. Missing or incomplete data were recorded as such and excluded from relevant data analyses. Data collection was carried out independently by the primary author, who ensured a uniform standard for data extraction. Data were sourced from original documents and captured as de-identified data in Microsoft Excel, stored securely on a password-protected device, thereby ensuring validity and confidentiality.

### Ethical considerations

Ethical clearance to conduct this study was obtained from the Human Research Ethics Committee of the University of the Witwatersrand (clearance certificate reference no. M220764) prior to the commencement of the study. As this was a retrospective medical records audit, informed consent from the patients’ guardians was not required.

## Results

A review of 150 outpatient referrals to the SRH STA department was conducted. Males were more commonly referred (*n* = 94, 62.7%). Younger children were referred more frequently than older children, with a decreasing frequency of referrals with increasing age. Approximately a quarter (26%) of the referrals were younger than 12 months and 7.3% were between 60 months and 72 months of age. Most referrals were received from the paediatric outpatient clinic, which receives referrals from the local PHC clinics (66.7%). The most common reason for referral was speech delay (*n* = 57, 38%) but only 20 (35.1%) of these children were referred below the age of 3 years (Table 1).

**TABLE 1:** Demographics, source of referrals and reason for referrals included in the audit of the Speech Therapy and Audiology department at the district state hospital (*N* = 150).

Variables	*n*	%
**Gender**		
Male	94	62.7
Female	56	37.3
**Age (months)**		
0 – < 12	39	26.0
12 – < 24	24	16.0
24 – < 36	30	20.0
36 – < 48	28	18.7
48 – < 60	18	12.0
60 – < 72	11	7.3
**Source of referrals**		
Paediatric outpatient clinic	100	66.7
Hospitals	35	23.3
Therapeutic services	12	8.0
Schools	3	2.0
**Reason for referrals**		
Speech delay	57	38.0
Developmental delay	22	14.7
Suspected ASD	10	6.7
Articulation difficulty	4	2.7
Suspected hearing impairment	2	1.3
Other	55	36.7
**Other reasons for referrals (*N* = 55)**		
Neonatal complications	28	-
Genetic	12	-
Neurological	10	-
Infections	3	-
Gastro-oesophageal reflux	1	-
Failure to thrive	1	-

ASD, autism spectrum disorder.

**FIGURE 1:**
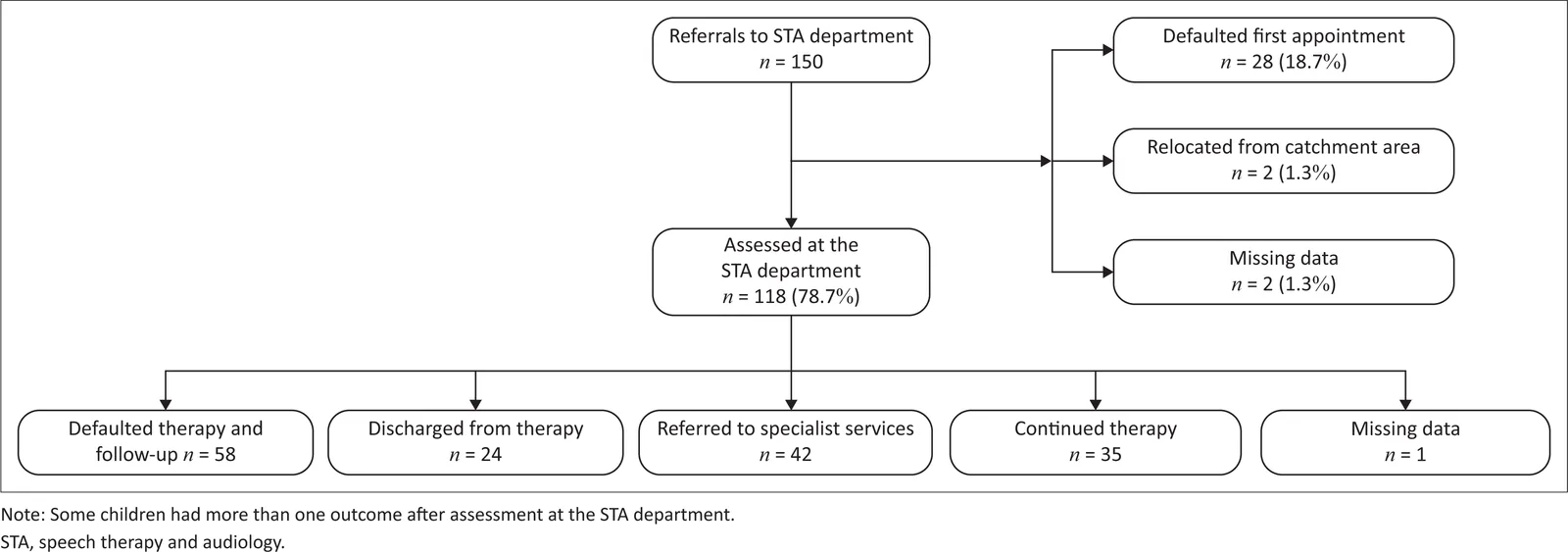
Flow diagram depicting the flow of patients before and after the initial assessment conducted in the Speech Therapy and Audiology department.

Only 78.7% of the referred children were assessed by the speech therapists and audiologists. Initial appointments were not attended by 28 (18.7%) patients, who were therefore not assessed. Two patients relocated outside the hospital’s catchment area and were referred to their geographically relevant STA clinics. In addition, two files were excluded from the audit because of extensive missing data (Figure 1).

As outlined in Figure 1, after the initial assessment of 118 children, 24 children (20.3%) were discharged from further follow-up, and 42 (35.6%) were referred for further evaluation by relevant specialised services. No follow-up data were available for 58 children (49.2%) because follow-up was defaulted after the initial assessment.

Of the children assessed, the majority were below the age of 3 years (*n* = 67, 56.8%). In addition, males constituted a predominant proportion (*n* = 76, 64.4%). The presence of maternal antenatal pregnancy-related comorbidities was recorded in 39% of the mothers of the assessed children. The most common recorded maternal comorbidity was HIV infection (41.3%), the second most common was gestational hypertension (23.9%), and 13.0% of the children assessed had been born from a twin pregnancy. The majority of the children were born at term (69.5%), and more normal vaginal deliveries occurred compared with birth by Caesarean section (49.2% vs. 38.1%) (Table 2). Children born preterm (18.6%) mostly had a gestational age (GA) between 32 weeks and < 37 weeks, one infant was born very preterm (28 weeks to < 32 weeks GA) and four extremely pre-term (less than 28 weeks GA). Birth weight was recorded within the normal range (2500 g – 3999 g) for 61.9%, and five neonates were macrosomic at birth (≥ 4000 g). The occurrence of delivery-related and neonatal complications was recorded in 86.4% of the assessed children, with 43.1% experiencing complications. One-and 5-min Apgar scores were recorded for 72% of the assessed children. Twenty per cent (*n* = 17) of the children with documented Apgar scores were recorded as having an Apgar score of ≤ 6 at 1-min and 7.1% (*n* = 6) at 5-min (Table 2).

**TABLE 2:** Demographic, maternal and perinatal history of children assessed at the Speech Therapy and Audiology department (*N* = 118).

Variables	*n*	%
**Age (months)**		
< 12	28	23.7
12 – < 24	18	15.3
24 – < 36	21	17.8
36 – < 48	24	20.3
48 – < 60	17	14.4
60 – < 72	10	8.5
**Gender**		
Male	76	64.4
Female	42	35.6
**Pregnancy history**		
Normal	58	49.2
Maternal comorbidity in pregnancy	46	39.0
Unknown or not documented or records incomplete	14	11.9
**Maternal comorbidity in pregnancy (*n* = 46)**		
HIV positive	19	41.3
Antepartum haemorrhage	2	4.3
HIV positive and antepartum haemorrhage	1	2.2
Gestational hypertension	11	23.9
Multiple pregnancy†	6	13.0
Prenatal alcohol exposure	1	2.2
Prenatal drug exposure	1	2.2
Other	5	10.9
**Gestation**		
Term	82	69.5
Preterm	22	18.6
Unknown or not documented or records incomplete	14	11.9
**Preterm gestational age (weeks)‡ (*n* = 22)**		
< 28/40	4	18.2
28 to < 32/40	1	4.5
32 to < 37/40	17	77.3
**Mode of delivery**		
Normal vaginal delivery	58	49.2
Caesarean section	45	38.1
Unknown or not documented or records incomplete	15	12.7
**Apgar score at 1 min**		
0–3	5	4.2
4–6	12	10.2
7–10	68	57.6
Born before arrival	3	2.5
Unknown	30	25.4
**Apgar score at 5 min**		
0–3	2	1.7
4–6	4	3.4
7–10	78	66.1
Born before arrival	3	2.5
Unknown	31	26.3
**Birth weight**		
≤ 999 g	3	2.5
1000 g – 1499 g	4	3.4
1500 g – 2499 g	20	16.9
2500 g – 3999 g	73	61.9
≥ 4000 g	5	4.2
Unknown	13	11.0
**Documented perinatal complications§ (*N* = 102)**		
Yes	44	43.1
No	58	56.9

HIV, human immunodeficiency virus.

† , Three mothers with multiple pregnancies had dual pathology;

‡ , WHO subcategories of preterm birth (World Health Organization, 2023);

§ , Perinatal complications at delivery included both delivery-related and early neonatal complications.

Children who were assessed, the primary home language data were recorded in 70 files, with English being identified as the primary home language in 61.4% of cases. Other home languages used included isiZulu (17.1%), Afrikaans (5.7%), and a combination of other official South African and non-South African languages, such as Lingala, Shona and French (15.7%). Most (87.1%) of the children were recorded as being bilingual. It was not recorded whether children spoke more than two languages.

Receptive and expressive language assessments were conducted using the Rossetti Infant-Toddler Language Scale and the Preschool Language Scale, Fifth Edition (Rossetti, 1990; Zimmerman et al., 2011). Assessment results were available for 96 (81%) children. Receptive language abilities were delayed between 12 months and 48 months, and 59.4% of those assessed experienced delays of more than 12 months. A greater number of children (67.7%) were assessed as having expressive language delay, ranging between 12 months and 49 months. Associations of risk factors for speech and language delay were analysed, and children from a bilingual household were not more likely to present with a receptive or expressive language delay of greater than 12 months (Table 3). No significant associations were found between the presence of maternal comorbidities during pregnancy and children with receptive language delay (OR = 1.28, *p* = 0.43) or expressive language delay (OR = 1.71, *p* = 0.23) of more than 12 months (Table 3).

**TABLE 3:** Receptive and expressive language delay associations.

Variables	*n*	%	Odd’s ratio	*p*-value
**Bilingual household**				
Receptive language delay > 12 months	44	72.1	0.35	0.21
Expressive language delay > 12 months	49	80.3	0.31	0.15
**Presence of maternal comorbidities**				
Receptive language delay > 12 months	22	47.8	1.28	0.43
Expressive language delay > 12 months	23	50.0	1.71	0.23

Audiology screening assessments, using distortion product otoacoustic emissions (DPOAEs) at 500 Hz, 1000 Hz, 2000 Hz and 4000 Hz and pure tone audiometry when indicated, were performed on 102 children, of which 88.2% had normal hearing (Table 4). Three children were confirmed with hearing loss and were referred. Fifteen children did not receive audiology screening as it was not indicated (*n* = 7) according to the Joint Commission of Infant Hearing (JCIH, 2007) or they defaulted their audiology appointment (*n* = 8).

**TABLE 4:** Audiology screening of children assessed at the Speech Therapy and Audiology department (*N* = 118).

Audiology screening	*N*	%
**Audiology screening done (*n* = 102)**		
Normal	90	88.2
Hearing loss	3	2.9
Inconclusive	9	8.8
**Audiology screening not done (*n* = 15)**		
Defaulted audiology screening	8	53.3
Audiology screening not indicated†	7	46.7
**Unknown or Missing records (*n* = 1)**	1	-

JCIH, Joint Commission of Infant Hearing.

† , No JCIH risk factors present to warrant screening.

Two children were referred to the STA department with suspected hearing impairment. Of these, one was among the three children who were ultimately diagnosed with hearing loss. The second child had inconclusive results and was referred for a sedation auditory brainstem response (ABR) assessment; however, subsequent follow-up was defaulted, and hearing loss could not be confirmed.

The three children diagnosed with hearing loss were all born at term. One child had an identified risk factor of neonatal jaundice and was referred for audiology assessment at 4 years of age. The second child with hearing loss had craniofacial dysplasia and was referred at the age of 1 year. The third child had no identified risk factors and was referred at age 2 years 11 months with delayed speech and suspected hearing impairment. Nine children were recorded as having inconclusive audiology results, but the hearing assessments could not be concluded as subsequent audiology appointments were missed.

Specialist referrals following the speech therapists’ and audiologists’ assessments were primarily to the NDC (*n* = 28, 66.7%). Two children required hearing assistive devices, and additional referrals included the Ear, Nose, and Throat specialist, auditory processing assessment, craniofacial clinic and plastic surgery services.

Of the 28 children referred for assessment at the NDC, 3 (11%) defaulted the NDC appointment. The most common diagnosis made by the NDC doctors was that of ASD (*n* = 13, 52%). Global developmental delay was diagnosed in six children, and developmental language delay in three children. Four children were found to be syndromic. Genetic syndromes were confirmed in two ASD patients, with one child diagnosed with Fragile X Syndrome. Atypical Rett syndrome was confirmed in one of the two female children diagnosed with ASD.

Ten children were referred with suspected ASD, of whom seven (70%) received a confirmed ASD diagnosis from the neurodevelopmental subspecialist. Of the remaining three children, one discontinued therapy at the hospital to seek therapy and neurodevelopmental consultation with a private practitioner, another defaulted on both therapy and the NDC appointment, and the third child was scheduled for an NDC appointment, which was outside the audit period. The mean age of referral to the STA department of the children ultimately diagnosed with ASD was 43.38 months (s.d. 12.13), and the mean age of ASD diagnosis for this sub-group, by the NDC, was 49.54 months (s.d. 11.47).

**FIGURE 2:**
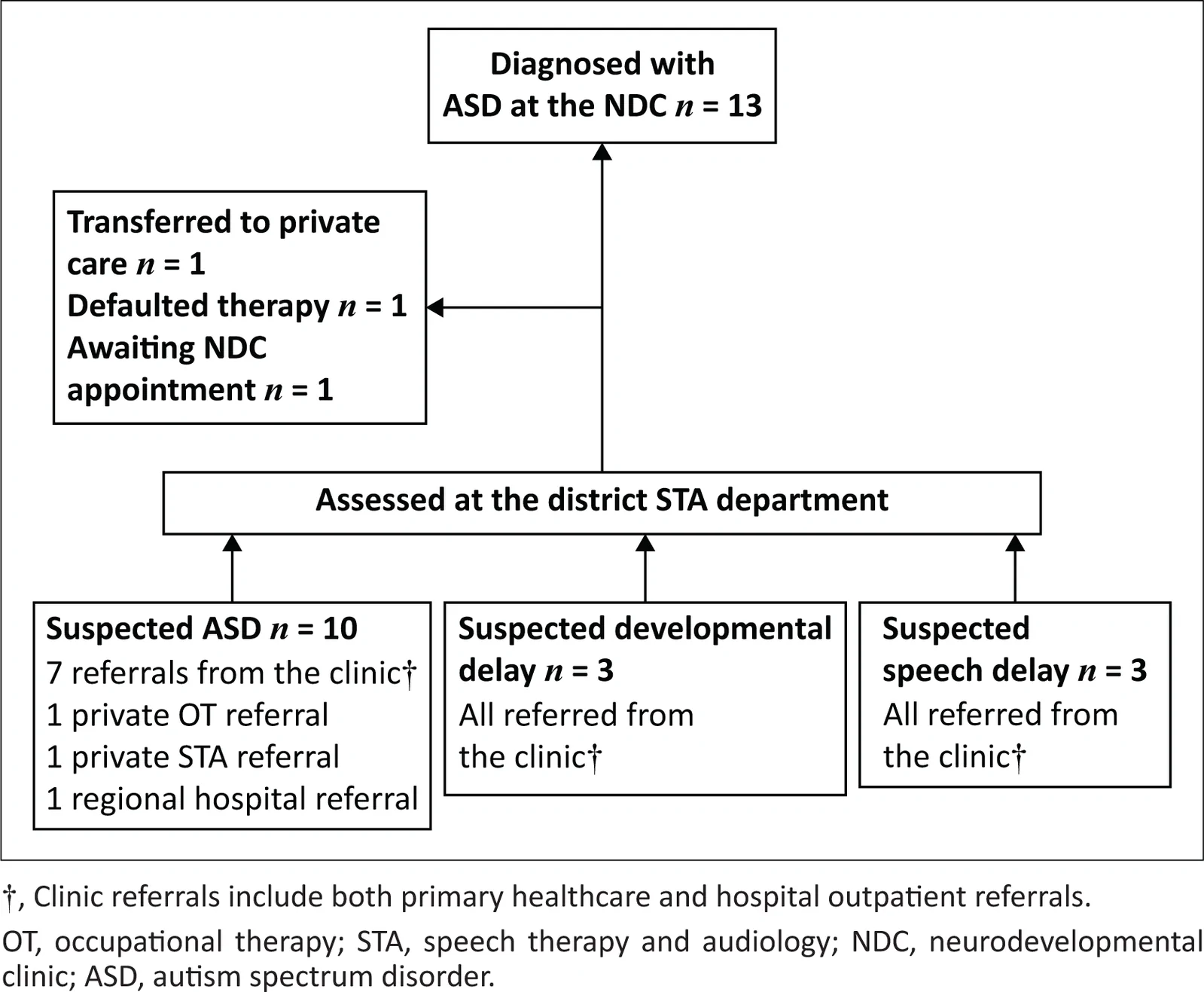
Flow diagram depicting the source of referrals and the initial suspected diagnoses for the 13 children diagnosed with autism spectrum disorder at the neurodevelopmental clinic.

Of the children diagnosed with ASD, just over half (*n* = 7, 53.8%) had initially been referred to the STA department with a presumptive diagnosis of ASD (Figure 2). Other presenting concerns prompting referral included speech delay (*n* = 3) and developmental delay (*n* = 3). The majority of those diagnosed with ASD were male (84.6%) (female to male ratio of 1:5.5), delivered at term (92.3%) with no family history of language delay (84.6%). There was no association found between a family history of language delay and a subsequent diagnosis of ASD (*p* = 0.64).

## Discussion

In this audit, we reviewed the records of 150 children referred to the STA department at a district-level hospital in Johannesburg to describe the referral process, clinical characteristics, risk factors and diagnostic outcomes. In addition, we examined associations between potential risk factors and receptive or expressive language delay.

Almost two-thirds of the children referred to the STA department were under 3 years old. These results are promising, as early identification and intervention for children with speech and language disorders are crucial for better outcomes in cognitive, scholastic and social development (Claassen et al., 2016; Schoeman et al., 2017; Vameghi et al., 2015).

Nearly 80% of children referred to the STA department attended the initial appointment, but less than one- third continued attending follow-up appointments after the initial assessment. However, these results must be interpreted in the context of the COVID-19 pandemic, which resulted in various levels of lockdown and restriction of non-essential services between March 2020 and 04 April 2022 (‘Statement by President Cyril Ramaphosa on the termination of the National State of Disaster in response to the COVID-19 pandemic – 04 April 2022’, 2022). During this period, the outpatient STA department’s services were temporarily halted as a measure to reduce the spread of corona virus infections (Balton et al., 2022). Patient apprehension about the contagious nature of COVID-19 led to the avoidance of hospital visits and the defaulting on scheduled appointments in SA and abroad (Almutairi et al., 2022; Balton et al., 2022). Other factors, such as the family’s socioeconomic status, compounded by the COVID-19 pandemic, may have contributed to the high number of children defaulting on appointments. In Uganda, parents of children with disabilities were consulted through telephonic interviews in May 2020 and July of 2020 on the impact of the COVID-19 pandemic on the accessibility of healthcare. All parents reported difficulties accessing healthcare and rehabilitative services as a result of lockdown restrictions, increased transport costs, and financial constraints stemming from reduced income during the COVID-19 pandemic (Mbazzi et al., 2022). Virtual tele-therapy is a well-utilised, flexible approach to therapy used by many speech-language therapists in high-income settings as an alternative to in-person rehabilitation services (Chadd et al., 2021); however, as in our cohort, tele-therapy is not widely utilised in LMICs. The high rate of defaulting follow-up therapy in our population may have been, in part, because of the lack of a feasible alternative to in-person therapy other than issuing a home programme at the district hospital.

Prior to the COVID-19 pandemic, Schoeman et al. (2017) identified factors contributing to defaulting therapy in a cohort of children living in an informal settlement in SA. The primary reason stated by parents was the prioritisation of physical well-being over the attainment of developmental milestones. Other factors, such as work commitments, transport difficulties, and forgetting appointment dates were also cited as reasons for defaulting on therapy (Schoeman et al., 2017). These findings highlight multiple contributors to therapy defaulting that were perhaps compounded by the pandemic, suggesting that the high rate of defaulting follow-up observed in our study may not have been solely attributable to our data collection occurring during the pandemic. Parents play a pivotal role in their children’s early childhood development and need to be informed of the importance of early diagnosis and timely intervention for children with possible developmental delays (Schoeman et al., 2017; Vameghi et al., 2015). Strategies employed to mitigate defaulting follow-up include appointment reminders and the provision of therapy closer to the patient’s place of residence, as logistic difficulties of travelling with a child with disability need to be considered (Bigna et al., 2014; Nota et al., 2015; Schoeman et al., 2017). The provision of holistic care in terms of a potentially easier referral path into specialist services at the hospital may have contributed to the retention of the children continuing in care.

While cultural and linguistic diversity defines the South African national context, with 11 official languages and widespread multilingualism, English emerged as the primary home language utilised. This contrasts with Statistics South Africa’s published data, indicating that English is used by only 8.7% of South Africa’s population and by 9.2% of the population residing in Gauteng province (Stats SA, 2022). Other non-South African languages, such as Lingala and Shona, were also recorded but were less prevalent in our cohort. This may point to the migrant population from Zimbabwe and Central Africa, which this district state hospital serves (Stats SA, 2022). Despite the prevalence of bilingualism among participants, our study found no significant association between children from bilingual homes and language delays. This resonates with the consensus that bilingualism does not adversely affect the speech and language development of neurotypical children (McLeod et al., 2016; Peña et al., 2011).

Biological risk factors have been shown to be strong predictors of language delay. Uzun Çiçek et al. (2020) identified a high rate of perinatal and neonatal risk factors in Turkish pre-school children diagnosed with intellectual disabilities and language delays. We found no associations between pregnancy-related comorbidities and language outcomes. These differences may be because of the different cognitive profiles of the two cohorts, as children in the Uzun Çiçek et al. (2020) study had intellectual disabilities, whereas our cohort exhibited a broader developmental profile.

The identification of medical causes of language delay, particularly hearing loss, should be prioritised when investigating potential aetiologies. This was pertinent to our study, as hearing loss was identified in three children. The Health Professions Council of South Africa (HPCSA) promotes Universal Newborn Hearing Screening (UNHS) for the timely identification and intervention of children with congenital hearing loss, which has a reported prevalence of 3–6 per 1000 live births in SA (Swanepoel & Störbeck, 2008). Although UNHS is currently not feasible in the SA healthcare sector because of resource constraints, targeted screening is recommended (Kanji & Khoza-Shangase, 2019).

The challenge with this approach is that a significant number of infants and children with hearing loss have no identified risk factors and remain undiagnosed until a substantial delay has occurred, affecting their developmental outcomes (Kuschke et al., 2020). This was evident in our audit, where one of the three children with no identifiable risk factors was diagnosed late with hearing loss. The other two children presented with risk factors for hearing loss, namely neonatal jaundice and craniofacial abnormalities. There is a need for increased awareness of children at risk of hearing impairment in the PHC setting.

Primary healthcare clinics are ideally situated within communities to serve as the first point of contact for developmental screening. The routine use of the RtHB, which contains a developmental screening section, equips the PHC nurse to identify children at risk of developmental delay and refer them timeously for more detailed assessments. However, there is a disparity between the ideal function of the PHC and the developmental screening being conducted. Petrocchi-Bartal and Khoza-Shangase evaluated 30 PHCs and found that only 30% of the clinics used the RtHB milestones to identify possible hearing impairment (Petrocchi-Bartal & Khoza-Shangase, 2016). Empowering healthcare workers at PHC facilities with the knowledge and skills to use the RtHB screening tool correctly could improve the identification and referral of infants and children who are not meeting their developmental potential.

Communication difficulties often serve as the first clinical indicator of broader developmental conditions, such as ASD. Although the exact aetiology of ASD is unknown, genetic and environmental factors have been implicated (Hodges et al., 2020; Salari et al., 2022). The prevalence of ASD is increasing partly as a result of increased awareness by both healthcare practitioners and the community (Salari et al., 2022). This was evident in our findings, as most of the children referred with suspected ASD as a cause of language delay were ultimately diagnosed with ASD by the NDC subspecialist. The early diagnosis and initiation of the required intervention in children with ASD will improve language development and cognition.

Autism spectrum disorder can be diagnosed as early as 18 months; however, a systematic review published in 2021 found the mean age at diagnosis for children ≤ 10 years of age to be 43.2 months (range: 30.9–74.7 months) (Van ’T Hof et al., 2021). The American Academy of Paediatrics advises developmental and autism-specific screening at various stages up to 30 months of age and beyond, and a referral for assessment if screening is positive (Hodges et al., 2020). As a result of limited ASD expertise and resources in SA, the diagnosis of ASD is likely occurring later than recommended. Data from an NDC in the Western Cape showed the mean age of diagnosis for children with ASD to be 53 months (range 42–68 months) (Rasdien et al., 2019), which reaffirms the need to increase awareness at the PHC level. Reassuringly, our mean age of ASD diagnosis was comparable to that reported by Van ’T Hof et al. (2021), underscoring that despite being situated at a district-level hospital, the ASD referral process resulted in timely diagnosis. The interdisciplinary NDC established at the hospital was the main factor resulting in prompt specialist consultation and timely ASD diagnosis. This NDC is part of a 10-year long outreach programme from a nearby tertiary academic hospital, which was set up to strengthen the services at the district facility and expedite the diagnostic process. The advantage of the NDC is the interdisciplinary cohesion of medical and therapeutic expertise, as each referred child is assessed simultaneously by an interdisciplinary team and an individualised care plan is formulated. There is regular communication between the team members, which further supports coordinated care for the patient. This approach also assists in creating a trusting environment for the patients and their parents or caregivers, as they are already familiar with the therapists prior to the NDC consultation. Having onsite subspecialist expertise contributed to an expedited diagnosis of ASD, as referred children would have otherwise been placed on a waiting list at the tertiary facilities, which may have resulted in older ages at diagnosis and possible defaulting of appointments.

The findings of this audit contribute to the understanding of referrals to speech therapy facilities at district-level hospitals and can help to guide the development of effective service utilisation. A child referred with speech and language delay may have an underlying aetiology that requires more urgent assessment and intervention, including children on the autism spectrum and children with hearing difficulties. The speech-language therapist or audiologist is often the first healthcare practitioner to assess children with more complex diagnoses, and proactive specialist referrals are essential for early diagnosis and intervention. Their ideal position between PHCs and the more specialised regional and tertiary facilities also serves to facilitate knowledge sharing with local healthcare providers, to promote awareness of developmental ‘red flags’ and the subsequent steps that should be taken.

A major limitation of our study was that the audit took place during the COVID-19 pandemic, which affected both the referrals and the follow-up of children at the STA department. We postulate that retention in care and improved follow-up would have occurred during a non-pandemic period. As this research formed part of a degree requirement, the study period could not be adjusted. The anticipated limitation of the inability to locate records, as well as missing information in individual files, was encountered in the retrospective file review. Importantly, the documentation of the child’s primary home language was missing from many files. The findings of this study only represent data from one district hospital in SA, and therefore, the results may not be generalisable.

## Conclusion

This audit provided insight into the referral process, clinical characteristics, risk factors, audiological findings, outcomes and follow-up care of children referred to a STA department at a South African district hospital.

The post-referral confirmation of speech and language delay, diagnosis of hearing impairment and subsequent referrals to specialist services emphasise the importance of early referral from PHC services. Further research is needed to investigate the reasons for the large number of children defaulting on the initial assessment as well as not continuing in therapy. Future research is also necessary to assess the burden of developmental disabilities, including ASD, GDD and hearing impairment in SA, which would assist in the strengthening of appropriate referral pathways for children presenting with speech and language delays. The presence of a specialist neurodevelopmental clinic at this district facility contributed to the timely diagnosis of ASD, and further collaborations between tertiary facilities and district facilities should be implemented across the healthcare sector.
